# Flavocytochrome *b*_2_-Mediated Electroactive Nanoparticles for Developing Amperometric L-Lactate Biosensors

**DOI:** 10.3390/bios13060587

**Published:** 2023-05-28

**Authors:** Olha Demkiv, Galina Gayda, Nataliya Stasyuk, Anna Moroz, Roman Serkiz, Asta Kausaite-Minkstimiene, Mykhailo Gonchar, Marina Nisnevitch

**Affiliations:** 1 Department of Analytical Biotechnology, Institute of Cell Biology National Academy of Sciences of Ukraine (ICB NASU), 14/16, Dragomanova Str., 79005 Lviv, Ukraine; demkivo@nas.gov.ua (O.D.); stasukne@nas.gov.ua (N.S.); morozanna1998@gmail.com (A.M.); rserkiz@gmail.com (R.S.); gonchar@cellbiol.lviv.ua (M.G.); 2 NanoTechnas—Center of Nanotechnology and Materials Science, Institute of Chemistry, Faculty of Chemistry and Geosciences, Vilnius University, Naugarduko 24, LT-03225 Vilnius, Lithuania; asta.kausaite@chf.vu.lt; 3 Department of Chemical Engineering, Ariel University, Kyriat-ha-Mada, Ariel 4070000, Israel

**Keywords:** L-Lactate, amperometric biosensor, flavocytochrome *b*_2_, electroactive nanoparticles

## Abstract

L-Lactate is an indicator of food quality, so its monitoring is essential. Enzymes of L-Lactate metabolism are promising tools for this aim. We describe here some highly sensitive biosensors for L-Lactate determination which were developed using flavocytochrome b_2_ (Fc*b*_2_) as a bio-recognition element, and electroactive nanoparticles (NPs) for enzyme immobilization. The enzyme was isolated from cells of the thermotolerant yeast *Ogataea polymorpha.* The possibility of direct electron transfer from the reduced form of Fc*b*_2_ to graphite electrodes has been confirmed, and the amplification of the electrochemical communication between the immobilized Fc*b*_2_ and the electrode surface was demonstrated to be achieved using redox nanomediators, both bound and freely diffusing. The fabricated biosensors exhibited high sensitivity (up to 1436 A·M^−1^·m^−2^), fast responses, and low limits of detection. One of the most effective biosensors, which contained co-immobilized Fc*b*_2_ and the hexacyanoferrate of gold, having a sensitivity of 253 A·M^−1^·m^−2^ without freely diffusing redox mediators, was used for L-Lactate analysis in samples of yogurts. A high correlation was observed between the values of analyte content determined using the biosensor and referenced enzymatic-chemical photometric methods. The developed biosensors based on Fc*b*_2_-mediated electroactive nanoparticles can be promising for applications in laboratories of food control.

## 1. Introduction

A healthy lifestyle is a popular trend in the modern world, which has prompted scientists working in the fields of medicine and the food industry to research ways to expand the range of healthy and functional foods. While they are not medicines, they can affect the person’s psychological or physiological state [1,2,3,4]. A special place in this category is occupied by dairy products: yogurt, cottage cheese, butter, buttermilk, kefir, koumiss, and others. These products have high nutritional value, being dietary and tasty, and are usually used to prevent and treat various gastrointestinal and other diseases. Dairy products are obtained from the milk of different animals via the action of lactic acid bacteria or other microorganisms that ferment carbohydrates, lactose in particular, into lactic acid [4,5,6].

The lactic acid anion L-Lactate (from now on—Lact), a nontoxic probiotic metabolite produced by lactic acid bacteria, plays an essential role in maintaining intestinal homeostasis and normal functioning, including providing human colon cells with metabolic energy sources [4,5,6,7]. Thus, eating foods containing significant amounts of Lact, such as yogurt, can substantially impact health by improving the composition of the microbiota [5]. That is why Lact is used in a wide range of foods and feeds, drinks, consumer goods, and products for healthcare. As an essential probiotic metabolite, Lact is an important analyte that must be monitored to assess the quality of foods and beverages [8,9,10].

For Lact analysis, many optic and electrochemical methods have been proposed [8,9,10,11,12,13,14,15,16,17]. Most of these methods are nonselective, time-consuming, costly, need pretreatment, and require expensive equipment. Enzymatic test systems and biosensors are promising instruments for Lact determination in industry and healthcare analytical laboratories. The available classic enzymatic methods of Lact analysis are generally using NAD^+^-dependent lactate dehydrogenase (LDH) from animal organs and bacterial lactate oxidase (LOX) [9,10,17,18,19,20,21,22]. Although LOX-based ABSs have excellent characteristics, especially if the enzyme is immobilized on the surface of 3D nano/microcarriers, their application is limited by the need for a second enzyme (peroxidase), native or artificial [21,22].

The yeast L-Lactate-cytochrome *c*-oxidoreductase (EC 1.1.2.3; flavocytochrome *b*_2_, Fc*b*_2_) is a promising biocatalyst in analytical methods for Lact determination, including biosensors [23,24,25,26,27,28,29,30]. Fc*b*_2_ from the baker’s yeast *Saccharomyces cerevisiae* is a homotetramer that contains two noncovalently bound cofactors, FMN and heme, per subunit [25]. Each subunit of Fc*b*_2_ is composed of two domains linked by a short hinge peptide: a C-terminal flavin-binding domain (or FMN L-Lactate dehydrogenase), which includes the active site for lactate oxidation and an N-terminal *b*_2_-cytochrome domain (or heme-containing domain), required for efficient cytochrome c reduction [25,26]. In experiments with the isolated, purified enzyme, the O_2_ molecules, instead of cytochrome c, become the subjects of reduction [26,31]. The enzyme exhibits absolute specificity to Lact, but the wide-scale application of Fc*b*_2_ from baker’s yeast in bio-analytics is limited by its instability and difficulties in enzyme purification [25,30,31,32,33,34].

The first reported Fc*b*_2_-based ABSs were developed using cells or cell lysates, from the yeasts *S. cerevisiae* and *Hansenula anomala* as the parental cells of this enzyme [30,31]. Many Lact-sensitive ABSs based on the recombinant cells have been proposed, especially in the last several years [32,33,34,35,36,37].

The highly purified thermostable Fc*b*_2_, isolated by Mykhailo Gonchar et al. twenty years ago from the cells of the thermotolerant methylotrophic yeast *Ogataea polymorpha* [38,39], was successfully used for the development of ABSs [27,28,39,40,41,42] and enzymatic–chemical methods for Lact determination [28]. The proposed analytical methods were successfully tested on real samples of food products, beverages, and biological liquids; these data are summarized in our earlier reviews [28,39].

The first ABS with Fc*b*_2_ of *O. polymorpha* was reported in 2005, and the possibility of direct electron transfer (DET) from the reduced form of this enzyme to a graphite electrode (GE) with a sensitivity of 1.1 A·M^−1^·m^−2^ was demonstrated [40]. The main disadvantage of this ABS was low sensitivity; even in the presence of the most electroactive mediator, phenazine methosulfate, the sensitivity value was 40 A·M^−1^·m^−2^ [40]. As efficient ways to improve the biosensors for Lact analysis, the methods of genetic engineering and/or nanotechnology were used [27,28]. Additionally, freely diffusing mediators were found to be effective amplifiers of a current signal in an ABS, due to efficient Fc*b*_2_-catalyzed oxidation of Lact [27,28].

Nanomaterials in the bio-recognition layer of the ABS serve as a matrix for enzyme immobilization and mediate electron transfer from the enzyme to the electrode surface. Nanoparticles (NPs) of metal oxides, metals, semiconductors, and composite NPs or nanohybrids perform various functions in electrochemical conversion circuits; in particular, much attention is paid to their mediator properties [10,11,12,13,14,15,16,17]. To improve electronic exchange between the surface of the working electrode and the enzyme in the planned ABS, a search for optimal nanomediators was performed.

The aims of our study were to develop highly sensitive ABSs for Lact determination using the thermostable yeast Fc*b*_2_ as a bio-recognition element, co-immobilized with electroactive nanomaterials and to demonstrate the applicability of the most sensitive reagentless ABS for Lact analysis in samples of commercial yogurts.

## 2. Materials and Methods

### 2.1. Reagents and Enzyme

Salts of transitional and noble metals, the sodium salt of L-Lactic acid, ascorbic acid, K_3_(Fe(CN)_6_), phenazine methosulfate (PMS), the Nafion solution, and all other reagents and solvents used in this work were purchased from Sigma-Aldrich (Steinheim, Germany). All reagents were of analytical grade and were used without additional purification. All solutions were prepared using ultrapure water.

L-Lactate-cytochrome c oxidoreductase (EC 1.1.2.3; flavocytochrome *b*_2_, Fc*b*_2_) was isolated from a cell-free extract (CE) of the thermotolerant methylotrophic yeast *Ogataea (Hansenula) polymorpha 356*, as described in detail earlier [39]. Briefly, yeast cells from the archived collection of microbial strains (ICB NASU, Lviv, Ukraine) were cultivated in flasks at 30 °C under intensive aeration in a mineral medium that contained 1% glucose, 0.2% sodium L-Lactate and 0.05% yeast extract. Freshly grown cells were collected via centrifugation, washed twice with water, suspended in a working buffer (50 mM phosphate buffer, pH 8.0), lyophilized, and kept at –20 °C until used. The dried cells were lysed with 10% *n*-butanol in a working buffer for 2 h at +4 °C. After the removal of cell debris via centrifugation (5000× *g*, 20 min, +4 °C), the supernatant was used as the CE for the isolation of Fc*b*_2_. The CE was put through a column with the anion exchange sorbent DEAE-Toyopearl 650 M (TSK-Gel, Kanagawa, Japan). Unbound proteins were washed with a buffer, and the Fc*b*_2_ was eluted with 15% ammonium sulfate (saturation at 0 °C) in a working buffer. Activity and protein concentration in each fraction were monitored. Fractions with the highest specific activity (16 U mg^–1^ of protein), for enzyme stabilization and concentration, were supplemented with ammonium sulfate (up to 70% saturation at 0 °C) and stored at −10 °C. L-Lactate standard solutions were prepared using the working buffer.

### 2.2. Synthesis of NPs

Bimetallic and trimetallic NPs were synthesized using the chemical reduction method [41]. Briefly, to obtain NiPtPdNPs (further—NiPtPd), solution 1 (H_2_PtCl_6_ and PdCl_3_) and solution 2 (NiSO_4_) were independently reduced by NaBH_4_. Then, solutions were mixed and supplemented with NaOH. PtZnNPs (further—PtZn) were synthesized from 10 mM H_2_PtCl_6_ using the Zn bath deposition method [42] with our modification [43].

To synthesize the hexacyanoferrates (HCFs) of Pt, Pd, or Au, 2 mL of 1 mM H_2_PtCl_6_, PdCl_3,_ or HAuCl_4_ solutions were first reduced by the addition of 0.2 mL of 100 mM ascorbic acid. After heating at 100 °C for 10 min under stirring, 8 mL of a 50 mM K_4_Fe(CN)_6_ solution was added and incubated for three days without stirring [44]. AgHCF and “green” gCu(II)HCF were synthesized as described earlier in [41,44], respectively.

The synthesized NPs were concentrated via centrifugation, washed with water, tested for pseudo-peroxidase activities, and stored at 4 °C [41].

Morphological analyses of the synthesized NPs were performed via scanning electron microscopy (SEM) [44].

### 2.3. Modification of Graphite Electrodes with Nanoparticles and Their Characterization

A graphite rod (GE, 3.05 mm diameter) was used as a working electrode. Modification of GEs with NPs and a study of their electrochemical properties were carried out as described earlier [41,44].

### 2.4. Development, Characterization, and Application of Bioelectrodes

To develop the Fc*b*_2_/NPs/GE, 5–10 μL of the Fc*b*_2_ solution was dropped onto the surface of the NPs/GE, air-dried, covered with Nafion, and stored as described in Section 2.3. The resulting bioelectrodes were studied in more detail as biosensors on Lact.

The most effective Fc*b*_2_/AuHCF-based ABS was used to determine Lact in the real samples of commercial yogurts.

The samples were tested using the graphical calibration method in a variant of the standard addition test (SAT) [41].

Each assay was performed in triplicate for two dilutions of the following yogurts: Lactel (“Lactalis”, Mykolaiv, Ukraine), Choodo (“Vimm-Bill-Dann”, Vyshneve, Ukraine), and Activia (“Danone”, Kherson, Ukraine).

### 2.5. Reference Method for Lact Determination in Real Sample

As a reference, the Prussian blue-based enzymatic–chemical method was used [28,43]. The principle of this method is the Fc*b*_2_-catalyzed oxidation of Lact in the presence of K_3_Fe(CN)_6_ (the enzymatic reaction). In this case, [Fe(CN)_6_]^3−^ is reduced to [Fe(CN)_6_]^4−^, which, when FeCl_3_ is added, forms a precipitate of Prussian blue (PB) (the chemical reaction). After the solubilization of the sediment, the concentration of the color product is detected via photometry or evaluated visually. The formation of a colloidal solution of PB indicates the presence of Lact, and the brightness of the color correlates with the Lact concentration.

Lact was analyzed in protein-free extracts of yogurts, described in Section 2.4. To obtain a protein-free extract, a 50% trichloroacetic acid solution was added to an aliquot of yogurt up to a final concentration of 10%; the solution was mixed, incubated for 30 min on ice, and centrifuged for 5 min at 10,000 rpm. The supernatant (S_n_) neutralized by NaOH was used for the determination of Lact with the described Fc*b*_2_/PB method.

The protocol is as follows: 30 μL of the S_n_ sample, first diluted 5-fold with water, was incubated for 30 min at 37 °C with 270 μL of the reaction mixture that contained 0.04 units/mL of Fc*b*_2_ and 3 mM K_3_Fe(CN)_6_ in a 50 mM phosphate buffer, at pH 8.0. Then, 100 μL of a 0.2 M FeCl_3_ solution in 30 mM HCl was added, and the formed precipitate was dissolved by adding 560 μL of 0.9 M oxalic acid. Optical density was determined with a SHIMADZU UV-1650 PC spectrophotometer at 680 nm, using the standard software “UV Probe 2.20” against a blank sample containing the phosphate buffer instead of Lact.

## 3. Results

### 3.1. Development of Fcb_2_-Based Amperometric Biosensors

**Figure 1:**
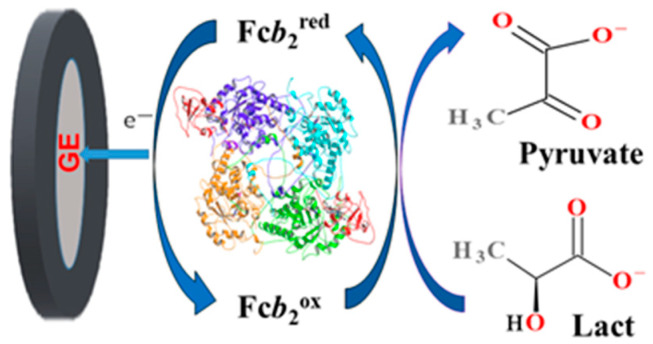
The principal scheme of Lact determination using the Fc*b*_2_-based ABS.

Fc*b*_2_ is a complex tetramer molecule containing a total of eight domains. Each domain is composed of heme and FMN, so the direct transfer of electrons (DET) is complicated, but it is possible. The ability of Fc*b*_2_ isolated from the cells of methylotrophic yeast *O. polymorpha* to achieve DET in an ABS was first demonstrated by our group [40]. It was concluded that the DET from the reduced form of Fc*b*_2_ to a graphite electrode (GE) takes place only for the molecules of the enzyme monolayer that are in direct contact with the surface of the GE. At the same time, the heme group must be correctly oriented at a specific distance from the electrode to achieve DET [40]. The scheme of DET in the ABS during the conversion of Lact in Fc*b*_2_-mediated catalysis is presented in Figure 1.

**Figure 2:**
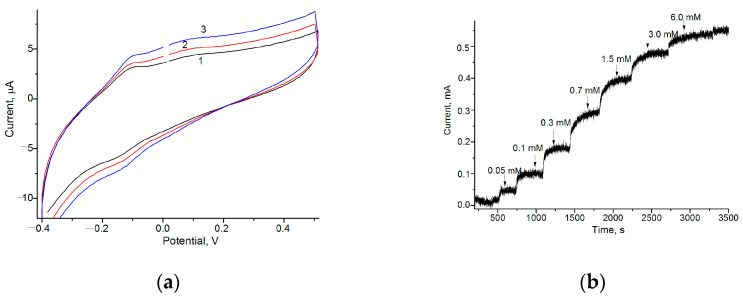
Amperometric characteristics of the Fc*b*_2_/GE as current responses to the increasing concentrations of Lact. (**a**) CV profiles at Lact concentrations: 0 mM (1, black), 2 mM (2, red), and 5 mM (3, blue); (**b**) chronoamperogram at the working potential of −75 mV.

To select the optimal working potential for the Fc*b*_2_-based ABS, the CV profiles for the Fc*b*_2_/GE in the presence and absence of Lact were compared. Figure 2a demonstrates increased oxidation and reduction peaks due to the addition of an analyte to the ABS.

According to the CV results, the peak of oxidation, as an output upon Lact addition, appeared in the range of −(150–50) mV. For further experiments, including a chronometric study, the potential of –75 mV was chosen as the optimal working potential.

### 3.2. Selection of the Optimal Redox Nanomediators and Their Properties

To enhance the effectiveness of electron transfer (ET) between the GE and Fc*b*_2_, the surface of the GE was modified with redox-active NPs as carriers for enzyme immobilization. These compounds, namely, NPs and hexacyanoferrates (HCFs) of noble and transition metals, were synthesized (see Section 2.2). Some of the previously obtained redox NPs, including the HCFs of Ag, Au, Pd, Pt, as well as NiPtPd, were characterized using CV in our previous papers [41,44,45] and are not described here.

Some NPs were used as artificial peroxidases (PO) or PO-like nanozymes in an arginine oxidase-based ABS [41] and as nanomediators for a laccase-based ABS [44]. Although the sizes of some studied materials did not satisfy the nanoscale criterion in all three dimensions, in our previous papers and here, we identify as NPs those materials whose nanoscale was confirmed using physical methods for at least one dimension [46].

**Figure 3:**
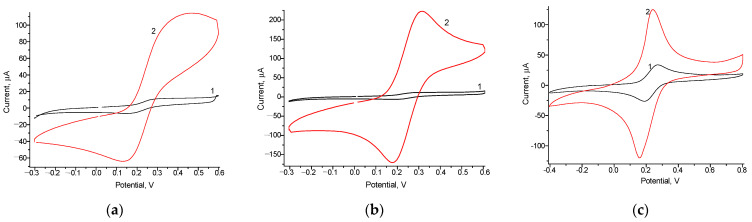
CV profiles of the control GE (**a**–**c,** line 1) and the modified electrodes: AuHCF/GE (**a**, 2), PtZn/GE (**b**, 2) and gCuHCF/GE (**c**, 2). Conditions: 10 mM K_3_Fe(CN)_6_, 100 mM KCl in 50 mM phosphate buffer, pH 6.5, and 20 °C.

**Figure A1:**
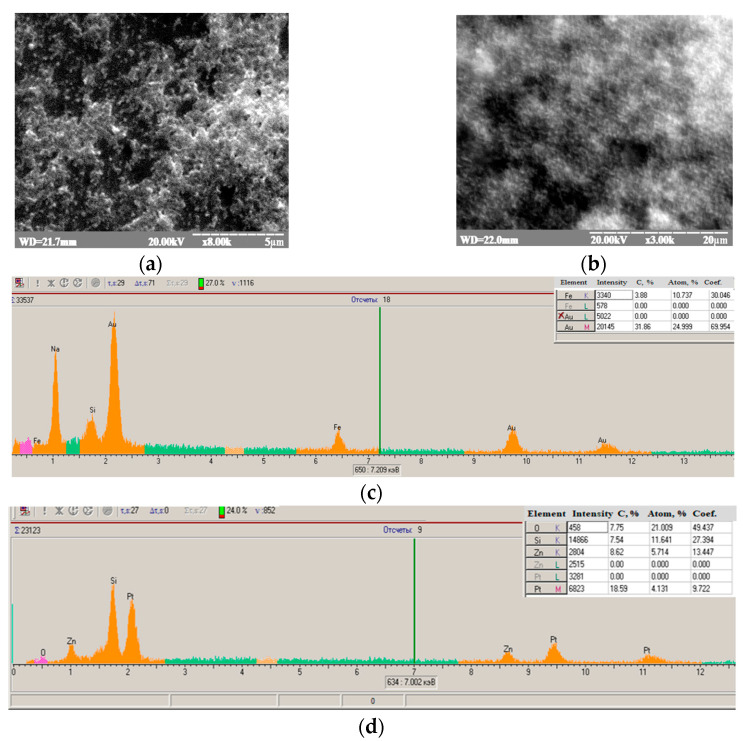
Structural and morphological characterizations of the AuHCF/NPs (**a**,**c**) and PtZn/NPs (**b**,**d**); the SEM images (**a**,**b**) with the correspondent XRM spectra (**c**,**d**).

Additionally, we searched for new NPs with high redox activity to use as prospective platforms for enzyme immobilization in the subsequent Fc*b*_2_-based ABS. For the AuHCF and the PtZn, detailed structural and morphological characteristics were analyzed (Figure A1) using the SEM-XRM approach. Redox properties of the GEs modified by NPs, and the control, were tested using CV (Figure 3) under the experimentally chosen optimal conditions [44].

According to the results of the CV study, the tested NPs were electroactive; the NPs/GEs had higher peaks of oxidation and reduction than those of the control GE (see Figure 3). All the studied NPs showed significantly increased electron transfer efficiency, making them very promising electroactive mediators for ABSs. Among them, the AuHCF/GE (Figure 3a) and Pt/GE (Figure 3b) were the most promising ones, having the highest redox activities.

### 3.3. Development of Fcb_2_/NPs-Based Amperometric Biosensors

The most active redox mediators coupled with Fc*b*_2_ were used to construct ABSs to ensure the MET between the enzyme and GE. According to the CV results for the Fc*b*_2_/GEs (Figure 2a), the peak of oxidation, as an output upon Lact addition, appeared in the range of −(150–50) mV. The optimal working potential for the study of the Fc*b*_2_/GEs and of the other proposed Fc*b*_2_/NPs/GEs was selected as –75 mV.

**Figure 4:**
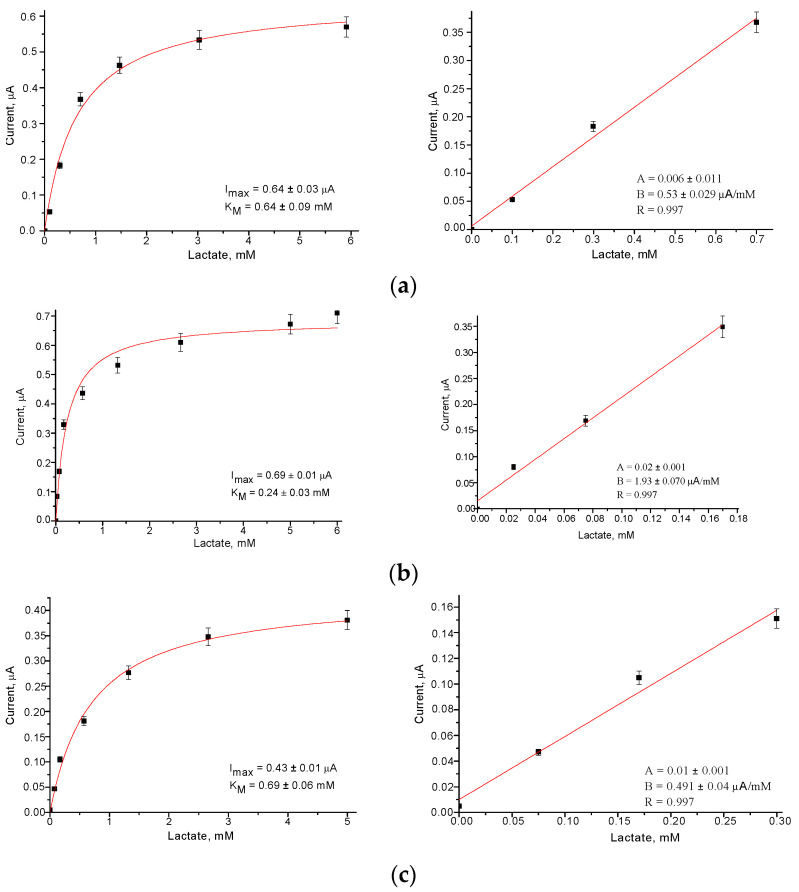

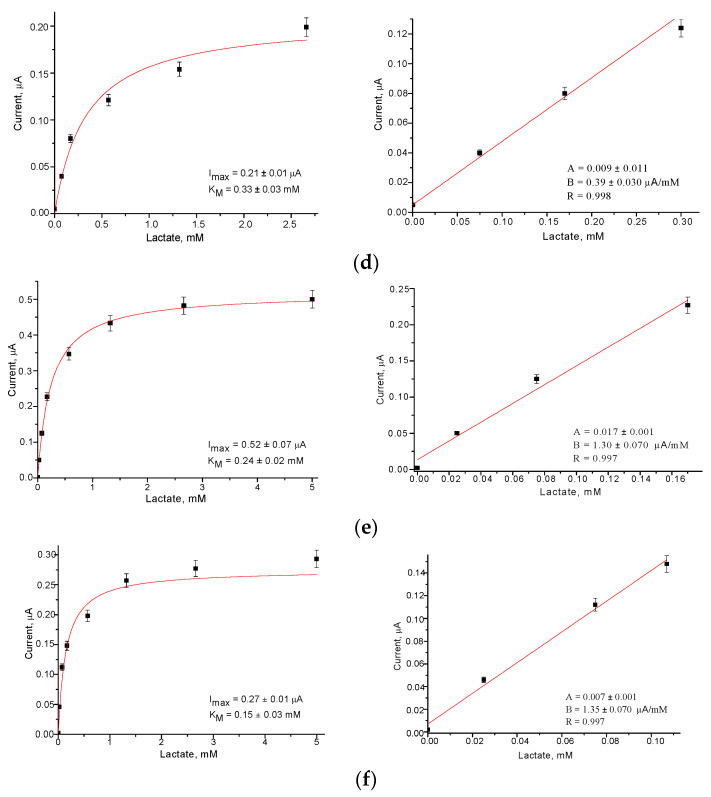
Calibration curves for Lact determination in wide (left) and linear (right) ranges using the Fc*b*_2_/NPs/GEs: (**a**)control, without NPs; (**b**–**f**) samples with AuHCF (**b**), PdHCF (**c**), PtHCF (**d**), PtZn (**e**), and NiPtPd (**f**). Each bioelectrode contained 25 mU of Fc*b*_2_ and 1 µg of NPs on the surface of the GE. Conditions: 50 mM phosphate buffer, pH 8.0, and working potential of −75 mV.

The calibration of the developed Fc*b*_2_/NPs/GEs was performed via a stepwise addition of the Lact solution, and the detailed chronoamperometric experiments are omitted here. The calibration graphs resulting from the corresponding chronoamperograms for the studied ABSs are presented in Figure 4.

The main operational characteristics of the ABSs, determined automatically from the calibration graphs (Figure 4) as described in our recent papers [41,44,45], are summarized in Table 1. Sensitivity was calculated as the ratio of the slope B value (from the linear regression graph) to the square of the active GE surface (7.3 mm^2^).

As can be seen from the presented results (Figure 4 and Table 1), the modification of the GE surface with PtZn, NiPtPd, and AuHCF led to an improvement in the operating parameters of the ABSs, namely, to an increasing sensitivity (2.5, 2.6 and 3.5-fold, respectively) and to a decreasing LOD (2.7, 4.3 and 2.7-fold, respectively), compared to the control Fc*b*_2_/GE. These ABSs may be useful for the analysis of Lact in food products, biological liquids, pharmaceuticals, and other real samples.

It is worth mentioning that NiPtPd is a PO mimetic and also demonstrates laccase-like activity [45]. Such properties of NPs may complicate our study and our understanding of MET processes. Therefore, for the detailed investigation of the influence of electroactive nanomediators on the effectivity of MET in the Fc*b*_2_/NPs/based ABSs, we selected the NPs of AuHCF and PtZn.

We concluded that the increased electron transfer in the Fc*b*_2_/NPs/GE may have been achieved thanks to NPs of a special shape and size. As Fc*b*_2_ is a large multidomain enzyme, it needs a matrix with small-size particles for immobilization and effective MET. SEM images proved that NPs of AuHCF and PtZn are really such materials (Figure A1). As a result, the Fc*b*_2_/AuHCF/GE and Fc*b*_2_/PtZn/GE demonstrated better operational characteristics in comparison with those of Fc*b*_2_/GE, due to MET without additional manipulation; that is, without using freely diffusing mediators.

### 3.4. Optimization of Lact Sensing for the Fcb_2_/AuHCF/GE

**Figure 5:**
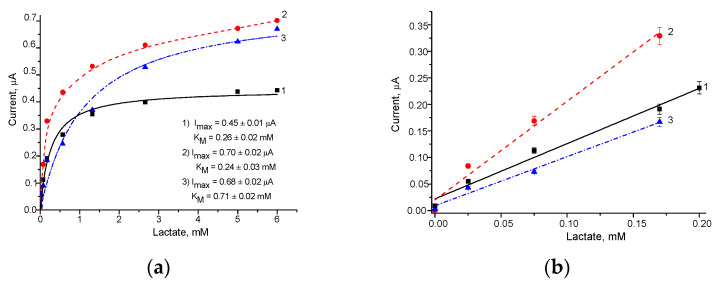
Calibration curves of the Fc*b*_2_/AuHCF/GEs in the broad (**a**) and linear (**b**) ranges of the dependence of enzyme quantity on the surface of GE; lines 1, 2, 3 correspond to 13, 25 and 50 mU of Fc*b*_2_, respectively. The working potential is −75 mV.

To improve the effectiveness of Lact sensing, the optimal quantities of Fc*b*_2_ placed on the surface of the AuHCF/GEs were experimentally chosen. The analytical properties of the resulting bioelectrodes were deduced from the graphs in Figure 5 and are summarized in Table 2.

According to the data presented in Table 2, the highest sensitivity (253 A·M^−1^·m^−2^) was achieved with 25 mU of Fc*b*_2_ on the GE surface in ABS-2. This sensitivity was 3.5-fold higher in comparison to that of the control GE without NPs in ABS-5, and 2-fold higher than that in ABS-3 and ABS-4, which contained 2-fold and 10-fold elevated quantities of Fc*b*_2_, respectively.

**Figure A2:**
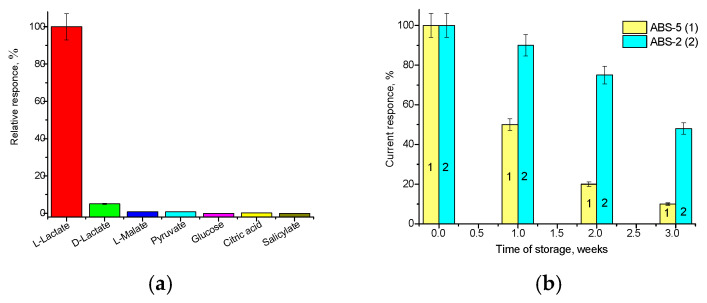
Properties of the Fc*b*_2-_based ABSs. The selectivity test for AuHCF/GE (ABS-2); current outputs in relative units (%) on the added compounds, up to 2 mM concentration. As a 100% signal, the highest current response on L-Lact was taken (**a**). Stabilities of the NPs-based ABS-2, and control ABS-5 without NPs (**b**).

**Figure A3:**
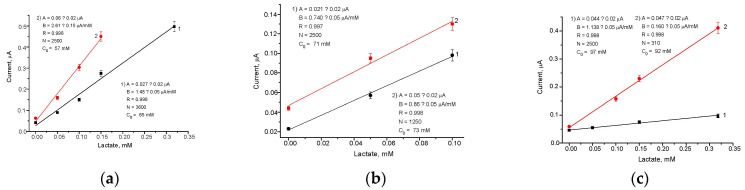
Assay of Lact in the samples of different yogurts (1–3) via graph SAT method (**a**–**c**, respectively), using Fc*b*_2_/AuHCF/GE (25 mU Fc*b*_2_ per ABS).

To characterize the Fc*b*_2_/AuHCF/GE in more detail, ABS-2 was chosen (see Table 2); the results are presented in Figure A3. To study the selectivity, ABS-2 was tested for its ability to respond to several individual natural substrates, namely, organic acids and glucose (Figure A2a). The stability outcomes of ABS-2 and ABS-5 kept under similar conditions (at +4 °C, over vapors of a working buffer) were compared (Figure A2b). According to the presented results, Fc*b*_2_/AuHCF/GE is highly selective (Figure A2a), and rather stable (Figure A2b). It is worth mentioning that co-immobilization of Fc*b*_2_ with AuHCF resulted in the enhanced stability of ABS-2, in comparison with ABS-5 without NPs.

### 3.5. Application of the Fcb_2_/AuHCF/GE for Lact Determination in the Real Samples

To demonstrate the applicability of the developed Fc*b*_2_/AuHCF/ABS for the determination of Lact, real samples of commercial yogurts were tested.

Lact is generated from lactose through a chain of enzymatic reactions. Cow’s milk contains 4 or 5% lactose. Lactose, being water-soluble, is associated with the whey portion of dairy foods. In the process of yogurt production, about 20% of the lactose present in milk is converted into lactic acid, and the Lact content in yogurt is about 0.9% or 100 mM [47]. Other fermented milk products, such as kefir, contain up to 2% or 220 mM Lact [47,48].

We carried out a determination of the Lact contents in strawberry yogurts. The results of the biosensor analysis using the graphical calibration method in a SAT variant [41,44] are presented in Figure A3 and summarized in Table 3. The numbering of the tested samples in Table 3 is the same as that in Figure A3.

The estimated average contents of Lact in yogurts corresponded to 60–95 mM, and such values are similar to the reported data (0.6–1.1%) [47,49]. The results of the quantitative analysis of Lact in the samples of yogurts using the Fc*b*_2_/AuHCF/ABS were compared with the data obtained using the enzymatic–chemical reference Fc*b*_2_/PB method (Table 3).

**Figure A4:**
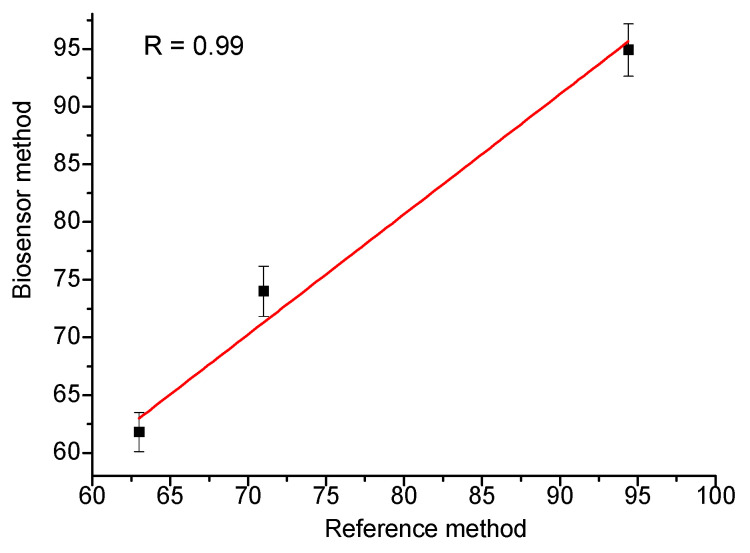
Correlation between the results of the determination of Lact content (mM) in drinking yogurts via reference enzymatic–chemical (Fc*b*_2_/PB) and biosensor methods.

The results of testing the effectiveness of the Fc*b*_2_/AuHCF-based ABS were shown to be within the permissible error limits (±10.0%). The results of Lact determination using both methods showed a reliably linear character with a strong correlation (R = 0.99), with differences of less than 5% (Figure A4). Thus, we demonstrated the applicability of the developed Fc*b*_2_/AuHCF/ABS for Lact analysis in yogurts.

### 3.6. Ways to Enhance the Sensitivity of the Fcb_2_-Based ABS

ABS sensitivity can be improved by increasing the rate/efficiency of DET and MET from the enzyme to the electrode. To satisfy this demand, close contact of the enzyme with the electrode surface must be ensured. The well-known general way to enhance the effectiveness of ET is the application of electroactive mediators—freely diffusing or/and immobilized ones coupled with the enzyme on the surface of the electrode [50].

Another way to enhance the sensitivity of an ABS is by selecting the optimal amount of the enzyme. Actually, according to the data of Table 2 and Table A1, the increased enzyme quantity on the surface of the Fc*b*_2_/AuHCF/ABS (more than 25 mU) caused decreasing ABS sensitivity. According to Table A1, a two-fold increase in enzyme quantity (from 250 to 500 mU) in Fc*b*_2_/ABSs led to a 2.9-fold decrease in sensitivity to Lact. Such results proved the importance of using optimal but not maximal quantities of the enzyme [44]. In other words, using the highest amount of the enzyme does not result in higher ABS sensitivity; this is probably related to the worsening diffusion process in the protein-enriched recognition layer.

**Figure 6:**
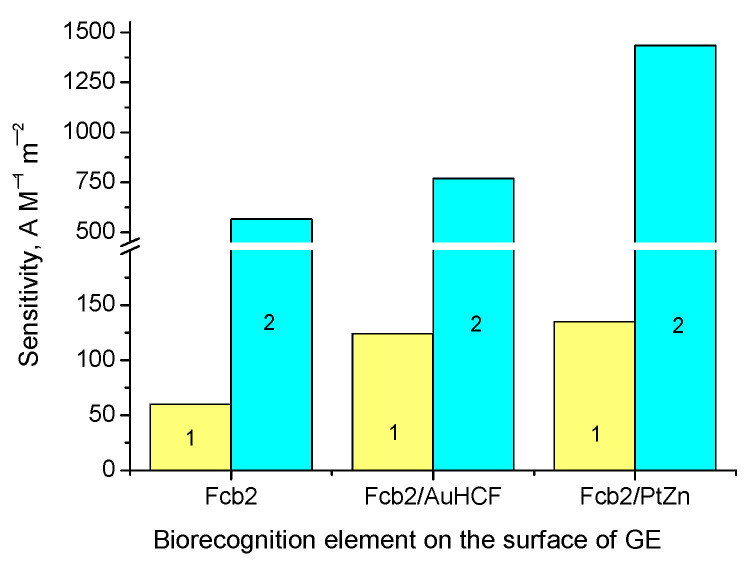
Impact of electroactive mediators, both immobilized (AuHCF and PtZn) and freely diffusing (PMS), on the sensitivities of the ABSs to Lact. ABSs with compositions Fc*b*_2_/GE, Fc*b*_2_/AuHCF/GE, and Fc*b*_2_/PtZn/GE were tested without PMS (1, yellow columns) and in the presence of PMS (2, cyan columns) in a 50 mM phosphate buffer, at pH 8.0, and at a working potential of −75 mV.

Additionally, we studied the impact of 1 mM PMS, one of the most electroactive freely diffusing mediators, on the analytical properties of the developed ABSs (Table A1 and Figure 6). Figure 6 demonstrates that the presence of PMS contributed to the improved sensitivity of the ABSs containing 250 mU of the enzyme.

As can be seen from the presented results (Table A1 and Figure 6), the addition of 1 mM PMS in the electrochemical cell caused increasing sensitivity in the Fc*b*_2_/GE, Fc*b*_2_/AuHCF/GE, and Fc*b*_2_/PtZn/GE (9.4-, 6.2- and 10.6-fold, respectively) compared to that of the corresponding bioelectrodes that were tested without PMS. The simultaneous impact of freely diffusing and co-immobilized mediators in NPs with the enzyme resulted in significantly enhanced sensitivity, due to the highly effective MET from the enzyme to the surface of the electrode. For example, the sensitivity of the Fc*b*_2_/PtZn/GE in the presence of PMS was 24-fold higher, in comparison to that of the Fc*b*_2_/GE without any mediator (Table A1).

## 4. Discussion

Fc*b*_2_ is a large ferrum-containing enzyme with a complex structure; its study is limited by protein instability. The Fc*b*_2_ successfully isolated by us from the thermotolerant yeast *O. polymorpha* was much more stable than the corresponding enzymes from the yeasts *S. cerevisiae* and *H. anomala*. The Fc*b*_2_ of *O. polymorpha* is stable enough to isolate, purify, characterize, lyophilize, and store for a sufficiently long time for use in developing analytical methods, including biosensors [28,39].

The possibility of DET from the reduced form of *O. polymorpha* Fc*b*_2_ to a GE was demonstrated earlier, as mentioned. It was reported that an optimal enzyme quantity is necessary to ensure its monolayer placement on the electrode’s surface and the appropriate orientation of hemes on the GE [40]. In a higher quantity, Fc*b*_2_ may form a multilayer structure on the GE surface, thus causing the limitation of DET and decreased sensitivity of the ABS. Additionally, generated pyruvate, a product of catalytic Lact oxidation gathered under a Nafion film, may inhibit Fc*b*_2_ as well [51,52].

We report here the fabrication and characterization of Lact-sensitive biosensors based on Fc*b*_2_ and electroactive NPs. All the proposed Fc*b*_2_/NPs/ABSs demonstrated improved operational parameters, compared to the control Fc*b*_2_/ABS. Specifically, the Fc*b*_2_/AuHCF/GE was 3.5-fold more sensitive to Lact in comparison to the control Fc*b*_2_/GE.

The analytical characteristics of other reported Lact-sensitive ABSs, compared to the ABSs proposed by us, are presented in Table A2.

It is worth mentioning that all the NPs reported here, and in our recent papers, are not only electroactive but catalytically active, too. They all possess pseudo-PO activity that allows them to decompose H_2_O_2_ in-solution and on amperometric electrodes. Being co-immobilized in a sensing layer with different oxidases, PO-like nanozymes positively impacted the analytical characteristics, especially the sensitivity of the developed ABSs, of catechol [44,51], ethanol, glucose, and arginine [41,51].

The following questions arise: What is the reason for the significant positive influence of PO-like nanozymes on the increased sensitivity of the Fc*b*_2_-based ABS? Is the electroactivity of the best NPs perhaps the only cause of such an effect? Unfortunately, the detailed mechanisms of heme-containing Fc*b*_2_ activity are still unknown [25,51,52,53,54,55,56,57,58,59,60]. Additionally, the structures and kinetic characteristics of Fc*b*_2_ from the yeasts *S. cerevisiae* and *H. anomala* have some differences [55,56,57], and the structure of Fc*b*_2_ from the yeast *O. polymorpha* is not yet reported. Perhaps this gap in enzymology is due to the lack of an enzyme available as a commercial product.

It can be concluded that the developed ABSs may be useful in the food industry and in other areas where precision analysis of Lact is necessary. It should be noted that the application of electroactive metallic NPs immobilized on the surface of the electrode can improve the sensitivity and stability of the Fc*b*_2_-based ABS.

## 5. Conclusions

In the current paper, a number of novel Lact-sensitive amperometric bioelectrodes based on oxidoreductase Fc*b*_2_ of thermotolerant methylotrophic yeast *O. polymorpha*, and the most effective nanomediators were developed and characterized. We investigated the enzyme-to-electrode ET process and showed that both DET and MET processes could be achieved by using bound NPs and low-molecular freely diffusing redox mediators. We found that the MET led to improved operational characteristics of Fc*b*_2_-based ABSs, especially to increased sensitivity. The main advantages of the proposed mono-enzyme ABSs are the simple architecture of the sensing layer and the ability to operate at low potentials in an oxygen-independent manner. The latter property of the developed Fc*b*_2_-based ABS is very valuable to ensure interference-free measurements and avoid the limitations inherent in LOX-based biosensors. The fabricated ABSs exhibited high sensitivities, fast responses, and low limits of detection. One of the most effective biosensors, Fc*b*_2_/AuHCF/GE, was used for Lact determination in commercial yogurts. A high correlation was observed between the values of Lact content determined using the biosensor and the reference methods.

## Figures and Tables

**Table 1:** The main operational parameters of the constructed Fc*b*_2_-based ABSs.

No	Nanomediator	Sensitivity, A·M^−1^·m^−2^	Linear Range, up to mM	LOD, mM	*I_max_*, µA	*K_M_^app^*, mM
1	–	72	0.70	0.05	0.64	0.64
2	PtHCF	53	0.30	0.05	0.21	0.33
3	PdHCF	70	0.30	0.02	0.43	0.70
4	gCuHCF	80	0.30	0.01	0.84	1.90
5	PtZn	178	0.18	0.02	0.52	0.24
6	NiPtPd	185	0.11	0.01	0.27	0.15
7	AuHCF	253	0.18	0.01	0.69	0.24

**Table 2:** Analytical properties of the Fc*b*_2_-based ABSs with different compositions.

No	ABS	Total Fc*b*_2_, mU	Sensitivity, A·M^−1^·m^−2^	Linear Range, up to mM	*I_max_*, µA	*K_M_^app^*, mM
1	Fc*b*_2_/AuHCF/GE	13	142	0.20	0.45	0.26
2	Fc*b*_2_/AuHCF/GE	25	253	0.17	0.70	0.24
3	Fc*b*_2_/AuHCF/GE	50	126	0.17	0.68	0.71
4	Fc*b*_2_/AuHCF/GE	250	124	0.35	0.48	0.10
5	Fc*b*_2_/GE	25	72	0.70	0.64	0.64
6	Fc*b*_2_/GE	250	60	0.16	0.23	0.44

**Table 3:** Lact content (mM) in the samples of yogurts estimated using two methods.

No	Yogurt	ABS Method	CV, %	Reference Method	CV, %
1	Lactel	61 ± 4.5	7.4	63 ± 4.0	6.3
2	Activia	72 ± 1.0	1.4	71 ± 5.4	7.6
3	Choodo	94.5 ± 2.5	2.6	95 ± 7.0	7.3

## Data Availability

The data are included within the present article.

## Appendix A

Appendix A presents the structural and morphological characteristics of the most effective nanoparticles (Figure A1), properties of Fc*b/*AuHCF/GE, namely, the selectivity and stability tests (Figure A2), Lact determination in the samples of yogurts using a biosensor (Figure A3), comparison of the Lact contents in yogurts analyzed with different methods (Figure A4), the effect of PMS on the main operational parameters of the ABSs (Table A1), and a comparison of the most effective Lact-sensitive ABSs (Table A2).

**Table A1:** The dependence of the main operational parameters of the ABSs on the presence of PMS.

ABS	Sensitivity, A·M^−1^·m^−2^	Linear Range, up to, mM	*I_max_*, µA	*K_M_^app^*, mM	Total Fc*b*_2,_ m-Units
Fc*b*_2_/GE	60	0.16	0.23	0.44	250
Fc*b*_2_/GE + PMS	565	0.08	1.70	0.30	250
Fc*b*_2_/GE + PMS	195	0.20	0.90	0.39	500
Fc*b*_2_/AuHCF/GE	124	0.35	0.48	0.10	250
Fc*b*_2_/AuHCF/GE + PMS	769	0.07	1.65	0.33	250
Fc*b*_2_/PtZn/GE	135	0.44	0.75	0.43	250
Fc*b*_2_/PtZn/GE + PMS	1436	0.12	2.74	0.18	250

**Table A2:** Comparison of the most effective Lact-sensitive ABSs.

Enzyme	Working Potential, V	Sensitivity, A M^−1^m^−2^	Linear Range, up to, mM	LOD, mM	Stability (days)	Reference
LOX	−0.1	5233	0.14	0.001	5	[22]
	0.15	7974	0.1	0.003	4.5	[21]
	0.3	450	3	0.05	* NR	[20]
	0.52	280	0.6	0.001	NR	[61]
	0.8	156	1.2	0.012	14	[62]
	0.6	400	0.35	0.032	NR	[63]
	0.15	NR	1	0.75 × 10^−3^	NR	[64]
	0.4	4300	0.7	0.022	30	[65]
** rFc*b*	0.25	48.6	2.7	0.3	NR	[28]
rFc*b*_2_	0.25	106	2	6	NR	[28]
rLDH	0	287.5	8	10	0.3	[37]
	0.1	638.3	6	14	0.9	
LDH	0.6	83	0.12	NR	7	[66]
	0.1	34.5	55	0.001	210	[67]
	0	34.6	10	0.1	NR	[68]
Fc*b*_2_	−0.75	565	0.08	0.015	5	This paper
		769	0.07	0.010	7	
		1436	0.12	0.010	7	

* NR—not reported; ** r—recombinant enzyme.
